# Prevalence of Hepatitis E genotype 3 among liver disease patients in Southwestern Nigeria

**DOI:** 10.1186/s12985-025-02989-z

**Published:** 2025-11-06

**Authors:** Olusola Anuoluwapo Akanbi, Adeolu Sunday Oluremi, Margaret Oluwatoyin Japhet, Folakemi Abiodun Osundare, Patrycja Klink, Dominik Harms, C.-Thomas Bock, Oluyinka Oladele Opaleye

**Affiliations:** 1 Department of Medical Microbiology and Parasitology, Ladoke Akintola University of Technology, Ogbomoso, Nigeria; 2 Department Infectious Diseases, Viral Gastroenteritis and Hepatitis Pathogens and Enteroviruses, Robert Koch Institute, Berlin, Germany; 3 Nigeria Centre for Disease Control and Prevention, Abuja, Nigeria; 4 Africa Centres for Disease Control and Prevention, Addis Ababa, Ethiopia; 5 Department of Microbiology, Obafemi Awolowo University, Ile-Ife, Osun Nigeria; 6 Institute of Tropical Medicine, University of Tuebingen, Tuebingen, 72071 Germany

**Keywords:** HBV, HEV, Coinfection, Liver disease, Nigeria

## Abstract

Owing to its high mortality rate, viral hepatitis is a major public health problem, especially in low-income countries. In Africa, hepatitis B virus (HBV) and hepatitis E virus (HEV) are highly endemic, and HBV/HEV coinfections, which are associated with more severe liver disease and poor outcomes, are common. HEV genotypes 1 and 2 have been associated with large human outbreaks, while 3 is known to circulate in pigs and sporadically in humans. In this study, the prevalence of HBV and HEV among individuals with acute or chronic liver diseases in Osun State, Southwest Nigeria, was analyzed. One hundred plasma samples from liver disease patients attending Ladoke Akintola University Teaching Hospital were analyzed for the presence of anti-HEV antibodies and hepatitis B surface antigen (HBsAg) via ELISA, and HEV RNA and HBV DNA were analyzed via RT‒PCR. Virus genotyping was performed by sequencing and subsequent phylogenetic analysis. Overall, 50 individuals (50%) were positive for HBsAg, of which 14 (28%) also tested positive for HBV DNA. Two individuals (2%) had occult HBV infection. Most HBV strains were genotype E, except for two genotype A (A2 and A3). Anti-HEV antibodies were detected in eight individuals (8%), with one (1%) being positive for anti-HEV IgM and seven (7%) for anti-HEV IgG. Nine (9%) samples had detectable HEV RNA, with one being HEV-3; a rare occurrence in Nigeria. Coinfection with HBV/HEV was detected in seven (7%) individuals. The prevalence of HEV in Nigeria is low, but considering the high prevalence of HBV and the possible complications due to HEV coinfection or superinfection, HEV screening and HBV vaccination targeting high-risk populations are emphasized.

## Background

In 2013, viral hepatitis was the leading cause of death worldwide, with hepatitis B virus (HBV) being the leading cause of death and hepatitis E virus (HEV) the leading cause of acute viral hepatitis worldwide [1]. Owing to widespread HBV vaccinations, the epidemiology of HBV is changing in many parts of the world, as seen in the reduction in the rates of infection in the Western Pacific, Southeast Asia, America, Europe and Eastern Mediterranean. Recent studies estimate the prevalence of HBV in Nigeria (5.4%−13.6%) to be declining making Nigeria an intermediate endemic region in contrast to previous high-endemic rating [Olakunde et al., 2025], however, in most African countries, the prevalence of HBV remains high [2]. Data on the prevalence and epidemiology of HEV on the African continent are insufficient to assess the true disease burden. However, recent studies suggest high exposure of the African population to HEV (Bagulo et al., 2020). HEV-1 and 2 seem to be restricted to infections in humans only being implicated in major outbreaks in poor resource settings, however, other genotypes 3–6, occur in other animals and poses major zoonotic risks. HEV-3 spread widely in pigs which has been identified as a major risk factor for HEV infection in humans in Southwestern, Nigeria [Oluremi et al., 2023]. Although HEV monoinfection tends to be mild, superinfection or coinfection with other viruses can present additional risks [3]. HEV superinfection or coinfection in patients with underlying chronic hepatitis B (CHB) can result in acute exacerbation of their liver disease. In patients with cirrhosis, a superinfection can result in decompensated liver disease and increased mortality rates.

Despite unclear mechanisms of interaction between these two conditions, HEV superinfection clearly alters the course of CHB disease into a detrimental pattern with poor outcomes [4]. Prevention of HEV infection, as well as its aggressive treatment in coinfected patients, might be an important strategy for reducing related morbidity and mortality, but the paucity of comprehensive clinical evidence hinders this approach. Although there is increasing evidence of HEV contamination in donated blood from qualified blood donors, in most African countries, routine testing of donor blood is restricted to HIV, hepatitis B, and hepatitis C virus (HCV) [5].

In areas where viral hepatitis viruses are endemic, the likelihood of dual infections is also high [3]. A study of viral hepatitis among liver disease patients in India reported that coinfection with HEV and HBV was the most common hepatitis virus coinfection [4, 5]. There is a lack of information on HEV infection and its coinfection rates with HBV in Nigeria. This study was therefore carried out to assess the seroprevalence and molecular characteristics of HBV and HEV, including genotype distribution and coinfection patterns among patients with suspected liver diseases in Southwest Nigeria.

## Materials and methods

### Study area and sample collection

Using convenience sampling, a total of 100 blood samples were collected from consenting adults attending the Gastroenterology Unit of LAUTECH Teaching Hospital, which included 43 chronic and 57 acute liver disease patients in 2019. The plasma was separated after centrifugation and stored at −20^°^C until analysis. Demographic and baseline data (including HIV status, liver function test results, and HCV status) were obtained for each participant.

Inclusion and exclusion criteria.

### Serology (HBsAg and anti-HEV)

Hepatitis B surface antigen (HBsAg) positivity was assessed via WantaiAiD HBsAg ELISA (Wantai, Beijing, China) following the manufacturer’s instructions.

Anti-HEV IgG and IgM antibodies were detected via ELISA via Wantai Hepatitis E Virus Diagnostics (Wantai, Beijing, China) according to the manufacturer´s instructions.

### Liver function tests

The plasma levels of liver enzymes (alanine aminotransferase (ALT) and aspartate aminotransferase (AST)) were determined via the colorimetric method of Reitman and Frankel [6, 7].

### Detection of nucleic acids and virus genotyping

Total nucleic acids were extracted from 140 µl of plasma via the QIAamp Viral RNA Kit (Qiagen, Hilden, Germany) on a QIAcube machine (Qiagen, Hilden, Germany) according to the manufacturer’s instructions. The eluates were collected and stored at −80 °C until use.

Real-time PCRs for HBV DNA and HEV RNA were carried out via virus-specific primers and hybridization probes on a LightCycler 480 instrument (Roche Diagnostics Corporation) as described previously [8, 9].

HBV DNA PCR amplification included nested PCR amplification of the partial preS/S region (332 bp) via previously described primer sets [8]. For the detection of HEV RNA and subsequent genotyping, all samples were analysed via two PCR assays: one-step reverse transcription (RT)-nested PCR and one-step RT-seminested PCR with generic primers targeting conserved ORF1 (307 bp) and ORF2 (401 bp) regions of the HEV (genotypes 1–4) genome, respectively, as described previously [9]. PCR was carried out via a Qiagen One-Step RT‒PCR Kit (Qiagen, Hilden, Germany) and HotStar Taq Master Mix (Qiagen, Hilden, Germany) in 25 µl total reactions. The PCR amplicons were visualized via 1.5% agarose gel electrophoresis and stained with GelRed (Biotium, Fremont, USA).

HBV and HEV genotyping were performed via direct sequencing of the nested PCR products as described, followed by phylogenetic analysis. Sequence electropherograms were edited and analysed via Geneious Prime 2020.0.5 software (https://www.geneious.com).

Sequence identities were confirmed via BLASTN, and multiple sequence alignments were generated via Geneious Prime 2020.0.5. Phylogenetic trees were constructed from generated alignments via the maximum likelihood algorithm based on the Tamura‒Nei model implemented in MEGA version 7.0 [10]. All laboratory analyses were carried out at the Robert Koch Institute, Berlin, Germany.

### Statistical analysis

The viral load was expressed in viral copy numbers per ml of plasma (copies/ml). Patient data are expressed as averages or percentages, and comparisons between groups were performed via the Mann‒Whitney U test, with 95% confidence intervals (CIs) and p values less than 0.05 considered statistically significant. Statistical data were analysed via GraphPad Prism 7 (GraphPad Software, San Diego, USA).

## Results

**Fig. 1:**
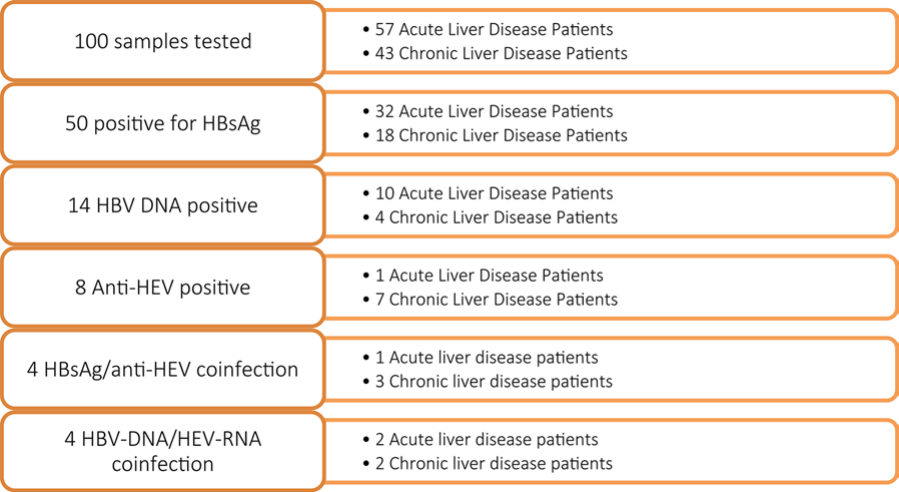
Flow diagram showing a summary of the number of patients tested and the detection outcomes

A total of 100 patients with liver disease participated in this study, including 57 individuals with acute liver disease and 43 individuals with chronic liver disease (Table 1, Fig. 1). The study cohort consisted of more male (*n* = 57) than female (*n* = 43) participants and had a median age of 38 years (IQR = 13.3). Median ALT and AST levels of 21.4 U/L (IQR = 10.7) and 25.9 U/L (IQR = 11.7) were detected, respectively. The mean plasma concentrations of ALT and AST were greater in patients with chronic liver diseases than in those with acute liver diseases, but the difference was not significant.

**Fig. 2:**
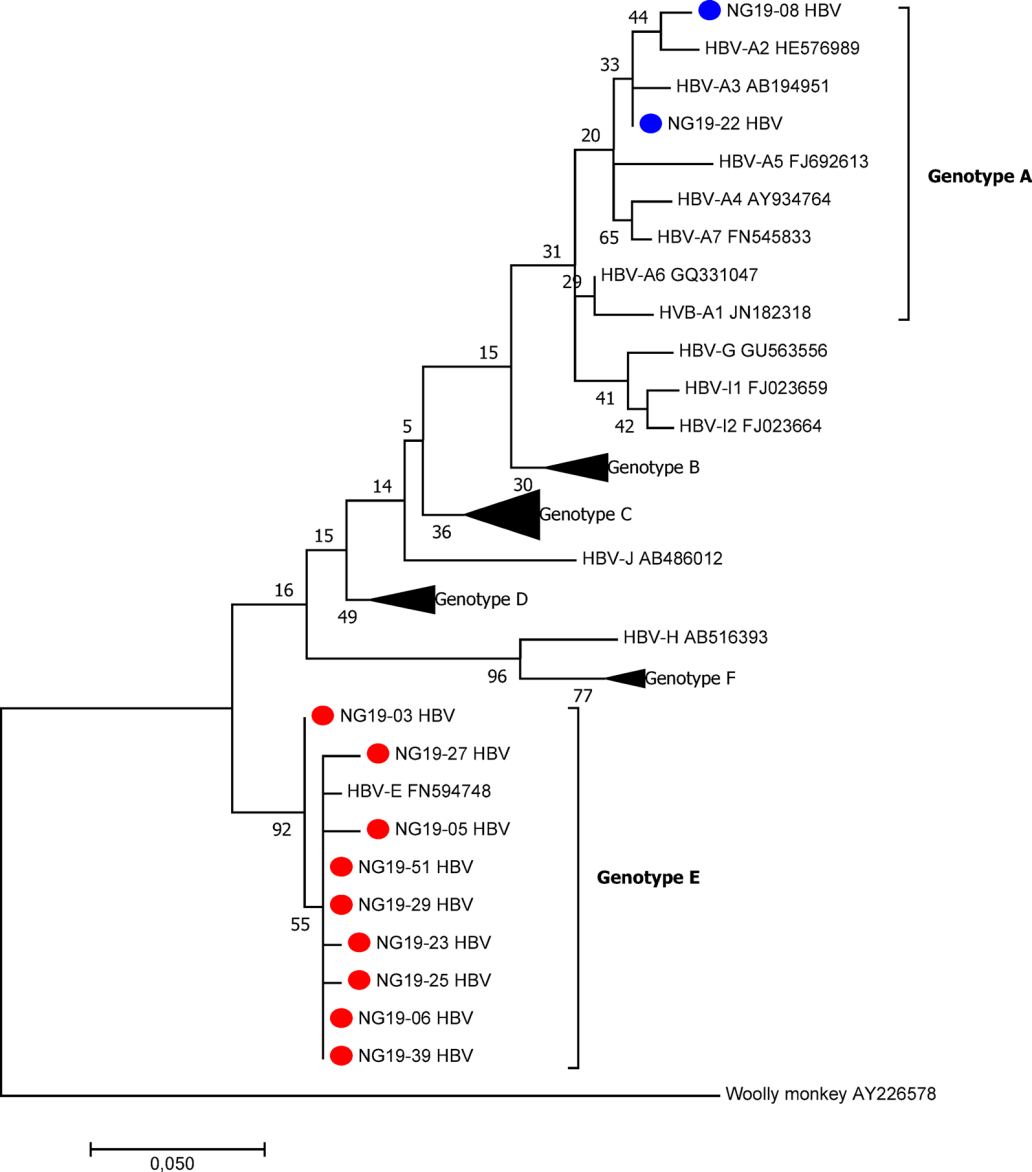
Phylogenetic tree constructed via partial coding of the S gene of HBV isolates (liver disease patients). The evolutionary history was inferred via maximum likelihood based on the Tamura‒Nei model. Phylogenetic reconstruction of the Pol region of sequences revealed that the HBV isolates clustered in the HBV-E branch, and the HBV sequences were compared with reference sequences representing 8 HBV genotypes (NCBI-GenBank accession numbers denoted). HBV isolates from this study NG19-XX (accession numbers PP983217–PP983227). Evolutionary analysis was conducted in MEGA 7.0.26

Among the 100 participants in this study, half (50%) were positive for HBsAg. Among these patients, 14 (28%) were positive for plasma HBV DNA, with an overall median viral load of 1.4 × 10^4^ copies/ml (IQR = 6.14 × 10^4^). The male-to-female ratio was 6:8, and most (71.4%) patients had acute liver disease. Among the 14 HBV DNA-positive samples, only eleven were used in the viral phylogenetic analysis, while three were excluded because of the low quality of the sequences. Phylogenetic analysis revealed that most HBV strains (*n* = 8/11) belonged to genotype E, whereas two belonged to genotype A (A2 and A3) (Fig. 2). Occult HBV infection (OBI), a condition in which patients test negative for HBsAg but have detectable levels of HBV DNA in plasma, was determined for two samples, both of which were found among chronic liver disease patients infected with the hepatitis B virus (HBV) genotype E.

**Fig. 3:**
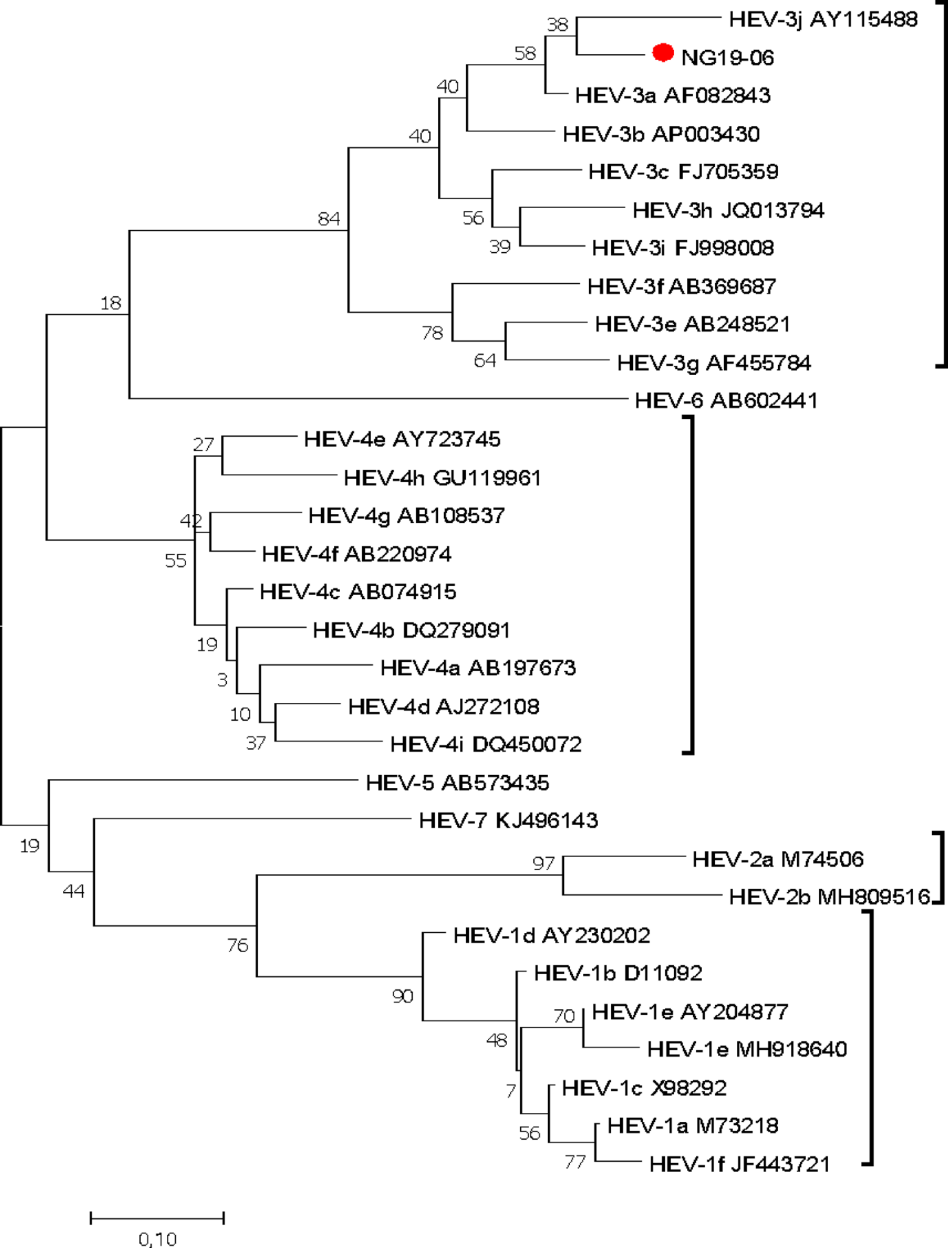
Reconstructed phylogenetic tree using the partial ORF 2 of HEV isolates (liver disease patients). The evolutionary history was inferred via maximum likelihood based on the Tamura‒Nei model. Phylogenetic reconstruction of the ORF2 region of the sequences revealed that the HEV isolate (NG19-06, Accession no. MT942691) clustered in the HEV genotype 3 branch, and the HEV sequences were compared with 30 reference sequences of HEV genotypes 1-7 (NCBI-GenBank accession numbers denoted). Evolutionary analyses were conducted in MEGA7.0.26

Anti-HEV antibodies were detected in eight samples (8%), with seven (7%) being IgG positive and one (1%) being positive for anti-HEV IgM. The IgM-positive sample was from a 46-year-old male with acute liver disease (Fig. 3).

Among the 100 participants, 9 (9%) had detectable HEV RNA in their plasma. Among these strains, three tested positive45 by qPCR (median HEV viral load of 1.78 × 10^3^ copies/ml; IQR = 7.22 × 10^3^), and 9 tested positive by RT‒PCR; however, only one was successfully sequenced and genotyped as HEV-3.

HBV/HEV coinfection was identified in 7 samples (7%). Four of the participants were positive for HBsAg/anti-HEV, three of whom were chronic liver disease patients. In four samples, HBV DNA and HEV RNA were detected; two samples were from individuals with chronic liver disease.

**Table 1:** Baseline data and detection of hepatitis viruses among study participants

Parameters	Acute liver disease patients	Chronic liver disease patients	*P* value	Total
Male/female	32/25	25/18		57/43
Median age (IQR)(years)	38 (13.2)	38 (13.7)	0.99	38 (13.2)
HBsAg positive (%)	32 (56.1)	18 (41.8)		50 (50)
HBV DNA positive (%)	10 (17.5)	4 (9.3)		14 (14)
Median HBV viral load (IQR)(copies/ml)	1.53 × 10^4^ (3.45 × 10^4^)	6.05 × 10^2^ (7.50 × 10^8^)	<0.01	1.40 × 10^4^ (6.14 × 10^4^)
HBeAg positive (%)	8 (14)	3(6.9)		11(11)
Anti-HCV (%)	0	0		0
Anti-HEV (%)	1 (1.8)	7 (16.3)		8 (8)
HEV RT‒PCR positive (%)	3 (5.3)	6 (13.9)		9 (9)
Median HEV copies/ml	8.29 × 10^3^	2.58 × 10^2^	< 0.01	1.78 × 10^3^(7.22 × 10^3^)
HBsAg/anti-HEV coinfection	1	3		4(4)
HBV-DNA/HEV-RNA coinfection	2	2		4(4)
HIV + ve (%)	1 (1.8)	10 (23.2)		11 (11)
Median ALT (IQR) (U/L)	21.4 (10.7)	21.6 (10.7)	0.94	21.4 (10.7)
Median AST (IQR) (U/L)	25.9 (11.7)	26.1 (11.7)	0.96	25.9 (11.7)

## Discussion

In this study, the anti-HEV seroprevalence was 8% among patients with a clinical diagnosis of liver disease, with 7% of the samples being positive for IgG and 1% for IgM. These results are in line with other studies from Nigeria, which generally revealed a higher prevalence of anti-HEV IgG than IgM [11]. However, the prevalence rates in Nigeria vary across study cohorts (6.5% among secondary school students from Kaduna, 13.4% in Ekiti state, 42.6% from pregnant women in Plateau state and 43% and 94% reported among health care workers and none-health care workers respectively in Ibadan, Oyo State [12] and are therefore not directly comparable.

HEV RNA was detected in 9% of the plasma samples. However, only one sample was successfully sequenced. Phylogenetic analysis revealed a relationship with HEV-3. The HEV patient strain identified in this study shares the highest nucleotide identity (92.0%) with a swine HEV strain (AF082843) isolated from pigs in the USA [13], which was shown to have evidence of cross-species infection. Buisson and colleagues described the circulation of HEV genotypes 1 and 3 in Nigeria [14], while we have also previously shown that HEV genotypes 1 and 2 circulated in Nigeria, as reported during the outbreak of acute hepatitis in 2017 in Northeast Nigeria [15, 16]. Multiple HEV genotypes are clearly in circulation in Nigeria in both human and animal populations.

There have been reports of high seroprevalence rates of HBsAg among patients with suspected liver diseases. In the present study, 50% of the participants were positive for HBsAg, 44% of whom were within the 31–40 years age group. A study in eastern Nigeria [17] reported a similar seroprevalence (50.7%). Genotypes of HBV have geographical variation in their distribution as well as implications for disease progression and response to antiviral therapy [18]. Genotype E is the most prevalent genotype in Nigeria and West Africa [19, 20], as found in this study. Here, HBV genotype A and subtypes 2 and 3 were also identified. HBV-A, although less common in Nigeria than does HBV-E, has been reported in previous studies [19] and other parts of Africa.

Occult HBV infection (OBI) is characterized by the absence of HBsAg, the presence or absence of anti-HBV antibodies and the presence of HBV DNA in the serum, making nucleic acid testing the most reliable laboratory method for the detection of OBI [8, 21]. An overall prevalence of 2.0% for OBI was observed in this study, with both samples being from female chronic liver disease patients. OBI has been previously reported in Nigeria, with varying prevalence depending on the region and cohort studied: 17% among blood donors [8], 11.2% among HIV patients [22], 8.7% among blood donors [23] and 5.4% among previously screened blood donors [24], indicating that OBI is frequently detected in Nigeria. Despite this, many health institutions and health facilities in Nigeria still depend only on serological methods for HBV diagnosis because of the cost implications and the lack of equipment for molecular testing. Although the prevalence of OBI in this study is lower than that in other reports, it nevertheless highlights the need for more nucleic acid testing for routine HBV diagnosis, which will help reduce the risk of HBV transmission via blood transfusion [24].

Superinfection or coinfection with HEV in chronic HBV patients or liver cancer patients can cause acute exacerbation of liver inflammation, decompensated liver disease and eventually increased mortality [3, 25]. In this study, overall, 7% (7/100) of the patients had evidence of coinfection with HBV and HEV, of whom five (71.4%) had chronic liver disease. Using ELISA, 4 (4%) plasma samples were found to be positive for HBsAg/anti-HEV, whereas 4 (5.0%) were positive by PCR for HBV DNA/HEV RNA; however, only one was positive by both serology and PCR. There is a lack of information on dual infection with HBV and HEV in Nigeria, but a study conducted among health care workers reported a prevalence of 27.3% [12]; this is higher than the results from this study, but this may be due to the different cohorts studied. In other parts of the world, HBV/HEV coinfection prevalence rates of 18% according to ELISA and 6.25% according to real-time PCR have been reported in India [26], while anti-HEV IgG and IgM seroprevalence rates of 28.5% and 1.7%, respectively, have been reported in persons with chronic HBV infection in the USA and Canada [27], and a study among patients with HBV-related liver diseases in Vietnam reported a high prevalence of HEV infection [4]. All these studies, including ours, emphasize the need to include routine HEV RNA screening, especially in high-risk groups such as HIV patients, pregnant women, chronic liver disease patients, immunocompromised patients, etc.

In this study, there was no significant difference in the serum levels of liver enzymes among patients with acute or chronic liver diseases. However, the group with chronic liver diseases presented slightly elevated levels than did those with acute liver diseases. This finding is not uncommon, as previous studies have shown that elevated liver enzymes (ALT) are not reliable predictors or markers of the extent or degree of liver damage [28] and therefore cannot be heavily relied upon, especially when initiating antiviral therapy. Reliance on elevated liver enzymes (ALT) may underestimate the true proportion of patients with normal or only minimally elevated ALT levels who have significant abnormalities in their liver tissue that can be detected only by a histologic examination [29].

## Conclusion

This study revealed high rates of hepatitis B virus (HBV) and hepatitis E virus (HEV) infection in patients with liver diseases in Nigeria and should be considered a high-risk group with respect to the risk of HEV infection. This study provides data on HBV/HEV coinfection in Nigeria, underscoring its public health importance and the necessity to incorporate HEV screening into the routine testing for liver diseases and more rigorous effort towards HBV vaccination. Additionally, the wide research gap in the study of HBV-HEV interactions and the link of HEV-3 infections in humans to zoonotic sources in Nigeria is worth addressing.

### Limitations

We were unable to obtain the whole-genome sequence of the HEV-3 isolates, and we could not sequence all positive samples due to their low viral load or sample degradation.

## Data Availability

HBV sequences obtained in this study have been deposited in GenBank under the accession accession numbers PP983217--PP983227. Other data will be available upon reasonable request.
